# 4-Hydroxy-2-nonenal Alkylated and Peroxynitrite Nitrated Proteins Localize to the Fused Mitochondria in Malpighian Epithelial Cells of Type IV Collagen* Drosophila* Mutants

**DOI:** 10.1155/2018/3502401

**Published:** 2018-01-30

**Authors:** András A. Kiss, Nikoletta Popovics, Zsolt Boldogkői, Katalin Csiszár, Mátyás Mink

**Affiliations:** ^1^Institute of Medical Biology, University of Szeged, Somogyi B. U. 4, 6720 Szeged, Hungary; ^2^John A. Burns School of Medicine, University of Hawaii, 1960 East West Road, Honolulu, HI 96822, USA

## Abstract

*Background*. Human type IV collagenopathy is associated with mutations within the* COL4A1* and to a less extent the* COL4A2* genes. The proteins encoded by these genes form heterotrimers and are the highest molar ratio components of the ubiquitous basement membrane. The clinical manifestations of the* COL4A1/A2* mutations are systemic affecting many tissues and organs among these kidneys. In order to uncover the cellular and biochemical alterations associated with aberrant type IV collagen, we have explored the phenotype of the Malpighian tubules, the secretory organ and insect kidney model, in* col4a1* collagen gene mutants of the fruit fly* Drosophila melanogaster*. In Malpighian epithelial cells of* col4a1* mutants, robust mitochondrial fusion indicated mutation-induced stress. Immunohistochemistry detected proteins nitrated by peroxynitrite that localized to the enlarged mitochondria and increased level of membrane peroxidation, assessed by the amount of proteins alkylated by 4-hydroxy-2-nonenal that similarly localized to the fused mitochondria. Nuclei within the Malpighian epithelium showed TUNEL-positivity suggesting cell degradation. The results demonstrated that* col4a1* mutations affect the epithelia and, consequently, secretory function of the Malpighian tubules and provide mechanistic insight into* col4a1* mutation-associated functional impairments not yet reported in human patients and in mouse models with mutant* COL4A1*.

## 1. Introduction

Basement membranes (BMs) are nanoscale sheets of extracellular matrices that play essential roles in multiple organs including muscle homeostasis, structures, and integrity of the dermal and ocular system, neuromuscular junctions, and blood filtration in the kidneys. The most abundant structural components of BMs include laminins, collagen IV, nidogens, perlecan, and agrin [1]. The ubiquitous mammalian BMs consist of heterotrimeric type IV collagens with (COL4A1)_2_COL4A1 composition. The clinical presentation of patients with* COL4A1 *mutation is systemic with numerous affected organs and tissues including the eyes, brain, the vascular system, skeletal muscles, and kidneys [2, 3]. A distinct form of type IV collagenopathy, Hereditary Angiopathy, Nephropathy, Aneurysms, and Muscle Cramps (HANAC) syndrome, is caused by N-terminal mutations within the* COL4A1 *gene. The renal manifestation of the same mutations in mouse models includes albuminuria, hematuria, glomerular cysts, and delays in glomerulogenesis and podocyte differentiation [4].

We have identified an allelic series of dominant, temperature-sensitive, antimorph mutations in the cognate* col4a1* gene of the fruit fly,* Drosophila melanogaster. *The col4a1^+/−^ heterozygotes are viable and fertile at permissive temperature of 20°C but die at 29°C. In these mutants, we have reported severe myopathy [5], tortuous BM, detachment of the gut epithelial and visceral muscle cells from the BM [6], intestinal dysfunction, overexpression of antimicrobial peptides, and excess synthesis of the oxidants hydrogen peroxide and peroxynitrite [7]. Peroxynitrite, by substituting the hydrogen atom by a nitro (-NO_2_) group adjacent to the hydroxyl group on the aromatic ring of tyrosine, adversely impacts protein functions and can be detected as a species-independent antigene [8]. Peroxynitrite can also remove a hydrogen atom from polyunsaturated fatty acids resulting in the formation of aldehydes, conjugated dienes, and hydroperoxyradicals that trigger a free radical chain reaction and membrane lipid damage by lipoperoxidation [9].

The main product of membranous polyunsaturated fatty acid peroxidation is the reactive 4-hydroxy-2-nonenal, HNE [10]. The reactivity of HNE with proteins relies on Michael addiction and, by modifying histidine residues, generates alkyl-conjugated polypeptides also detectable as species-independent antigens [11]. As there is no direct laboratory test to estimate lipid peroxidation, measurements of HNE-conjugated protein levels currently serve as surrogates [12]. The bulk of peroxynitrite reacts rapidly with carbon dioxide, present at ~1 mM in cells, forming the unstable product nitrosoperoxycarbonate (ONOOCO_2_^−^), one-third of which decomposes into carbonate (CO3^−*∙*^) and NO_2_^∙^ radicals and two-thirds into the neutral NO_3_^−^ and CO_2_ [13].

Insect Malpighian tubules serve as secretory organs. These renal tubules lack a vascular blood system and float freely in the hemocoel (blood-filled body cavity). The tubules are surrounded by BM and consist of two epithelial cell types, the metabolically active principal and the intercalated stellate cells [14]. The insect renal system is aglomerular, and urine is formed by active transport rather than by selective reabsorption of ultrafiltrate as in vertebrates. While the insect tubule system represents an intermediate towards the glomerular kidney, it fulfills the same basic functions of transport, excretion, and osmoregulation [15].

We have recently shown that the* col4a1 Drosophila *mutants develop stress fibers in their Malpighian cells and aberrantly express cell-surface integrin receptors [16]. In the present study, we have extended our research to address altered posttranslational protein modifications by peroxynitrite and 4-hydroxy-2-nonenal in the Malpighian tubules. The* col4a1 *mutants demonstrated heavy protein tyrosine nitration and protein-histidine alkylation that localized to the enlarged and fused mitochondria as signs of mitochondrial stress. HNE-protein adducts colocalized with the cytoplasmic membrane that was accompanied by cell degeneration in the tubules performing TUNEL-positivity, collectively suggesting that these aberrant processes are integral parts of* col4a1*-associated pathology.

## 2. Materials and Methods

### 2.1. Maintenance of* Drosophila* Strains

Wild-type* Oregon* flies and* col4a1* mutant stock with the* DTS-L3* allele were maintained at 20°C and 29°C on yeast-cornmeal-sucrose-agar food, consisting of nipagin to prevent fungal infection. The mutant stocks were kept heterozygous over the* CyRoi* balancer chromosome. Malpighian tubules were removed under carbon dioxide anesthesia from adults that were grown at both permissive and restrictive temperature for 14 days. Dissected Malpighian tubules were fixed in 4% paraformaldehyde dissolved in phosphate buffered saline (PBS) for 10 min, washed three times in PBS, permeabilized for 5 min in 0,1% Triton X, dissolved in PBS, and washed three times in PBS. Blocking was achieved in 5% BSA dissolved in PBS for 1 hour and washed three times in PBS.

### 2.2. Immunostaining and Antibodies

Nuclei in the dissected Malpighian tubules were counterstained by 1 *μ*g/ml 4′,6-diamino-2-phenylindole (DAPI) in 20 *μ*l PBS, 12 min in dark. F-actin was stained by 1 unit Texas Red™-X Phalloidin (ThermoFisher) in 20 *μ*l PBS for 20 min. A-Mannopyranosyl and a-glucopyranosyl residues as membrane markers were stained by Concanavalin A, Alexa Fluor™ 594 Conjugate (ThermoFisher) in 20 *μ*l PBS for 1 hour. We used 1 *μ*l mouse monoclonal anti-3-nitrotyrosine [39B6] (Abcam) in 20 *μ*l PBS for 1 hour and stained 4-hydroxynonenal conjugate by 1 *μ*l mouse monoclonal anti-4-hydroxynonenal antibody (Abcam) in 20 *μ*l PBS for 1 hour. Primary mouse antibodies were visualized by 1 *μ*l F(ab′) 2-Goat Anti-Mouse IgG (H + L) Cross Adsorbed Secondary Antibody conjugated with Alexa Fluor 488 (ThermoFisher) in 20 *μ*l PBS for 1 hour and 1 *μ*l Goat Anti-Mouse IgG (H + L) Cross Adsorbed Secondary Antibody, Alexa Fluor 350, in 20 *μ*l PBS for 1 hour. Mitochondria were visualized by the mitochondrially targeted EYFP (mito-GFP) following appropriate crosses [17].

### 2.3. Confocal Microscopy

Photomicrographs of the Malpighian tubules were generated by confocal laser scanning fluorescence microscopy (Olympus Life Science Europa GmbH, Hamburg, Germany). Microscope configuration was the following: objective lens: UPLSAPO 60x (oil, NA: 1.35); sampling speed: 8 *μ*s/pixel; line averaging: 2x; scanning mode: sequential unidirectional; excitation: 405 nm (DAPI), 543 nm (Texas Red), and 488 nm (Alexa Fluor 488); laser transmissivity: 7% were used for DAPI, 42% for Alexa Fluor 488 and 52% for Texas Red.

### 2.4. TUNEL-Labelling

Terminal deoxyribonucleotide transferase-mediated dUTP-fluorescein conjugate nick end labelling (TUNEL) was carried out by using the in situ cell death detection kit (Roche) as recommended. Embryos of mutant and control flies were incubated at 20°C or 29°C and L3-stage larvae collected. Nuclei in the Malpighian tubules were counterstained by 1 *μ*g/ml 4′,6-diamidino-2-phenylindole (DAPI). Labellings were visualized by a Hund-Wetzlar fluorescence microscope by using FITC or DAPI filters.

## 3. Results

### 3.1. Heavy Protein Nitration in* col4a1* Mutants

We have previously demonstrated that the* col4a1* mutant flies synthesize peroxynitrite at higher concentration as part of their antimicrobial immune response under restrictive conditions [7]. Peroxynitrite is produced by the diffusion-driven reaction of nitric oxide (NO) in the presence of oxidants such as the mitochondrial-derived radical superoxide anion, O_2_^∙−^. The sources of NO are at extramitochondrial sites and the dissolved gas diffuses into mitochondria, reacts with O2^*∙*-^, and disrupts protein functions by protein tyrosine nitration [18]. We therefore expected accumulation nitrated proteins in the mitochondria of* col4a1* mutant flies following incubation at 29°C.

**Figure 1:**
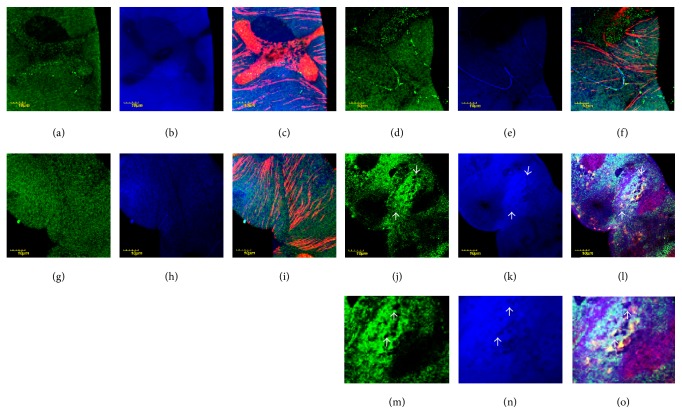
Protein nitration in Malpighian epithelial cells. Color code: mitochondria, green; nitrated proteins, blue; actin, red. (a) Wild-type flies incubated at 20°C, mitochondria, (b) nitrated proteins, and (c) merge. Note a stellate cell in (c). ((d), (e), (f)) Wild-type flies incubated at 29°C and displayed in the same order. ((g)–(i)) Mutant flies, incubated at 20°C, and ((j)–(l)) mutant flies, incubated at 29°C. Photomicrographs are displayed in the same order as in the upper row. Localization of nitrated proteins to mitochondria is shown in (c), (i), (f), and (l). Uneven distribution and fusion of mitochondria are demonstrated in (j). White arrows in (j), (k), and (l) pointing the region displayed in higher magnification in (m), (n), and (o). White arrows in (m), (n), and (o) showing regions with no/few mitochondria and the lack of staining, demonstrating localization to mitochondria with nitrated proteins indirectly.

We did not observe gross alterations in the Malpighian tubules of the mutants compared to control flies; mitochondria were distributed evenly in the cytoplasm and the fluorescent light intensities used to record nitrated proteins in the mutants were comparable to the control animals (Figures 1(a)–1(c) and Figures 1(g)–1(i)), following incubation at permissive condition. However, under restrictive conditions (29°C), we noted marked differences in the Malpighian tubules of mutant flies. While mitochondria in the epithelial cells of wild-type Malpighian tubules remained evenly distributed with no shape alteration at this temperature (Figure 1(d)), in mutants, mitochondrial fusion and uneven distribution were observed (Figure 1(j)). The level of nitrated proteins was remarkably higher in mutants in comparison with the control (Figure 1(k) versus Figure 1(e)) and these signals localized to the mitochondria (Figures 1(f) and 1(l)).

### 3.2. High Levels of Alkylated Proteins in the Mutants

**Figure 2:**
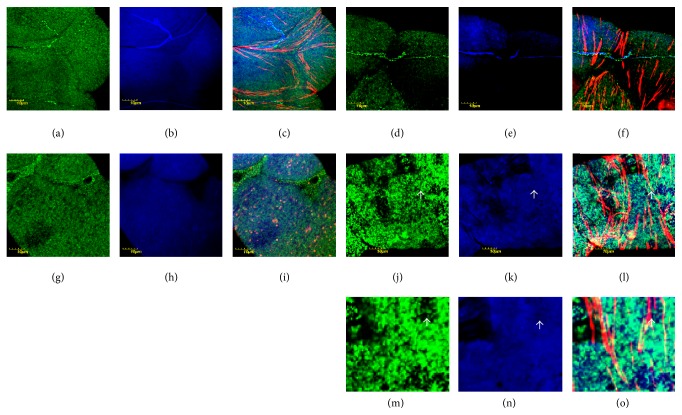
Protein-HNE adducts in Malpighian epithelium. Color code: mitochondria, green; protein-HNE adducts, blue; actin, red. (a) Mitochondria of wild-type flies incubated at 20°C, (b) protein-HNE adducts, and (c) merge. ((d), (e), (f)) Wild-type flies incubated at 29°C, shown in the same order. ((g)–(i)) Mutant flies, incubated at 20°C, and ((j)–(l)) mutant flies, incubated at 29°C. The order of photomicrographs is as in upper row. Note mitochondrial fusion in (j) and actin stress fibers in (l). White arrow in (j), (k), and (l) showing the portion displayed in higher magnification in (m), (n), and (o). White arrow in (m), (n), and (o): point regions with no/few mitochondria and the lack of staining, demonstrating localization of alkylated proteins to mitochondria indirectly.

The level of lipid peroxidation was determined indirectly by the accumulation of HNE-protein adducts. Results showed comparable amounts of alkylated proteins in the epithelial cells of mutant Malpighian tubules at permissive condition (Figure 2(b) versus Figure 2(h)), and the appearance of mitochondria remained unaffected in both mutants and controls under these conditions (Figure 2(a) versus Figure 2(g)). In mutants under restrictive conditions (29°C), uneven distribution and fusion of mitochondria occurred (Figure 2(j) versus Figure 2(d)), the mutants produced more HNE-protein adducts (Figure 2(k) versus Figure 2(e)), and the alkylated proteins localized to mitochondria (Figures 2(f) and 2(l)).

### 3.3. Protein-HNE Adducts Associate with Cytoplasmic Membrane

**Figure 3:**
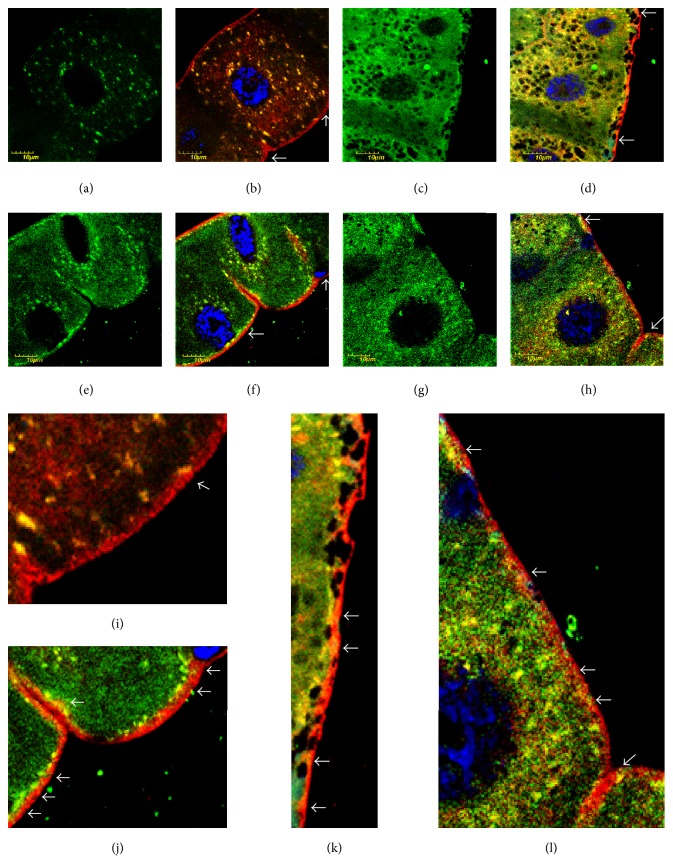
Cytoplasmic membrane-associated HNE-modified proteins in mutants. Color code: protein-HNE adducts, green; cytoplasmic membrane red; nuclei, blue. (a) Protein-HNE adducts in wild-type flies incubated at 20°C. (b) Overlay with membrane staining. (c) Protein-HNE adducts in wild-type flies incubated at 29°C. (d) Merged with membrane staining. ((e), (f), (g), (h)) Representative mutant, incubated at 20 or 29°C presented in the same order as in upper row. White arrows in (b), (d), (f), and (h). Regions presented in higher magnification in (i), (j), (k), and (l), respectively. White arrows in (i), (j), (k), and (l) show association of the cytoplasmic membrane with alkylated proteins. Note the notorious infiltration of HNE-modified proteins into the membrane in (l), which occurs at a less extent in (k). The membrane of wild-type animals is free of alkylated proteins (i), while they associate closely with the membrane in the mutant (j), incubated at permissive temperature.

We next explored the involvement of the cytoplasmic membrane in* col4a1*-associated pathology. We recorded numerous alkylation sites in form of punctate staining in colocalization with the cytoplasmic side of the membrane and apparent perinuclear accumulation in the Malpighian epithelial cells in the mutants at permissive conditions (20°C) (Figures 3(e) and 3(f)). This staining pattern was amplified upon shift to restrictive temperature (29°C) and the HNE-conjugated proteins appeared within the cytoplasmic membrane indicating direct membrane damage by lipid peroxidation (Figures 3(g) and 3(h)). In the control flies the cytoplasmic membrane remained intact and protein-HNE adducts appeared in the vicinity of the membrane at both permissive and restrictive conditions (Figures 3(a)–3(d)).

### 3.4. Cell Degeneration Detected by TUNEL-Positivity

**Figure 4:**
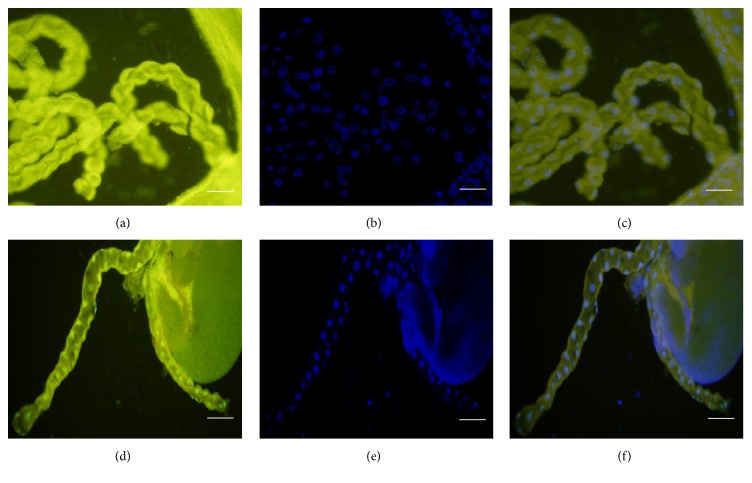
Fluorescence micrographs demonstrating TUNEL-positivity in Malpighian tubules. (a) TUNEL-staining of a Malpighian tubule of* col4a1* ^+/−^ L3-larva incubated at 20°C; (b) DAPI-staining; (c) merge, tubules appearing TUNEL-negative. (d) TUNEL-positive Malpighian tubule of a* col4a1* ^+/−^ L3-larva incubated at 29°C; (d) DAPI; (e) merge. Scale bars: (a)–(c) 50 *μ*m, (d)–(f) 100 *μ*m.

The epithelial cells of the Malpighian tubules proved to be TUNEL-positive in mutants at 29°C (Figures 4(d)–4(f)), but not at 20°C (Figures 4(a)–4(c)). These observations further supported our earlier observations of cell death affecting multiple tissues in* col4a1* mutants.

## 4. Discussion

*Drosophila* models provide useful tools for determining the pathomechanistic details, functional alterations, and some of the genotype-phenotype correlations of human monogenic disorders [19] including mutations associated with disorders of the kidneys as some of the human genes known to be associated with inherited nephrotic syndromes play conserved roles in renal functions from flies to humans [20]. There are nephrotic manifestations of human* COL4A1* mutations of the Hereditary Angiopathy, Nephropathy, Aneurysms, and Muscle Cramps (HANAC) syndrome [21] and recent research revealed glomerular hyperpermeability and adult onset glomerulocystic kidney disease in association with* COL4A1* mutations [4]. Some of the mechanistic elements in context of type IV collagen mutations, such as oxidative stress, have also been demonstrated [22]. However, evidence for chronic inflammation and posttranslational protein modifications are scarce and so far demonstrated only in* Drosophila col4a1 *mutants [7, 16].

Mitochondrial fusion occurs under situations of cellular stress. Merging of the contents of partially damaged mitochondria is interpreted as a complementation mechanism rescuing impaired organelles and function [23]. Our prior results demonstrated signs of cellular stress in the form of actin stress fibers in the Malpighian epithelial cells of* col4a1* mutant* Drosophila* [16]. Results of the current study show that mutation-associated stress induced mitochondrial hyperfusion also occurs under restrictive condition with the enlarged organelles unevenly distributed within cells resulting either in areas apparently lacking mitochondria or in organelle-enriched areas. A further consequence of* col4a1 *mutation is the accumulation of nitrated and alkylated proteins in the mutants that localize to both normal and fused mitochondria.

This observation indicates a peroxynitrite-mediated nitrosative stress in* col4a1* mutants that produce peroxynitrite at higher concentration [7]. We thus suggest that the elevated peroxynitrite level likely causes excess protein tyrosine nitration; however, this reaction does not deplete peroxynitrite in* col4a1 *mutants. Indeed, the still available peroxynitrite can initiate membrane damage by lipid peroxidation producing HNE, which in turn alkylates proteins by the mechanism of Michael addiction [8]. Direct association of alkylated proteins with the epithelial cell membrane of mutant Malpighian tubules supports this scenario. Furthermore, the mutation-induced stress directs the epithelia towards degeneration as demonstrated by the TUNEL-positivity of the nuclei.

**Figure 5:**
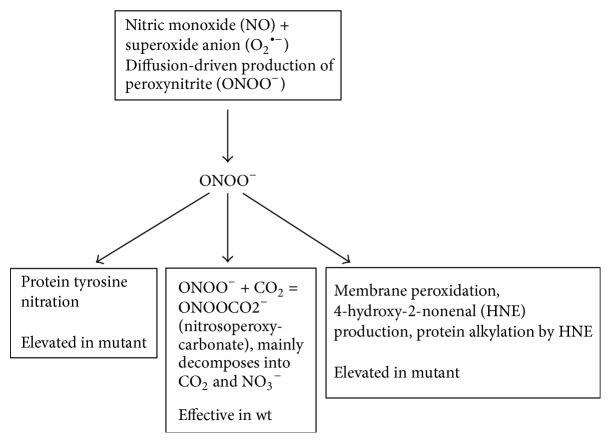
Schematic representation of peroxynitrite effects in wild-type flies shifting towards the neutralizing nitrosoperoxycarbonate pathway and in* col4a1* mutants towards protein nitration and alkylation involving membrane peroxidation.

The data presented here strongly suggest a central role for peroxynitrite in* col4a1*-associate defects. In wild-type animals and under physiological conditions, the nitrosoperoxycarbonate pathway is the preferential reaction of peroxynitrite, as the main decay products of nitrosoperoxycarbonate, nitrate anion, and carbon dioxide do not exert protein or membrane modification effects (Figure 5) [13]. In mutants, however, peroxynitrite is present above physiological concentrations, and it produces excess protein tyrosine nitration and forms HNE leading to protein alkylation, lipid peroxidation, membrane damage, aberrant mitochondria, epithelial cell death, and Malpighian tubule dysfunction.

## 5. Conclusions

*Drosophila* with* col4a1 *mutation synthesize peroxynitrite as a part of their stress response above physiological concentrations. The excess peroxynitrite triggers heavy protein tyrosine nitration and protein alkylation adversely affecting protein function; it also initiates membrane lipid peroxidation and mitochondrial fusion. In control animals, these posttranslational protein modifications remain at physiological levels by utilizing the nitrosoperoxycarbonate pathway to neutralize peroxynitrite. We suggest that the posttranslational protein modifications detected in the* col4a1 *mutant* Drosophila *model are integral parts of* col4a1*-associated pathology and represent pathomechanistic details that have not yet been addressed in human or mouse* COL4A1* mutants.
